# ECOPHAGE: Combating Antimicrobial Resistance Using Bacteriophages for Eco-Sustainable Agriculture and Food Systems

**DOI:** 10.3390/v15112224

**Published:** 2023-11-08

**Authors:** Pilar García, Rafael Tabla, Hany Anany, Roberto Bastias, Lone Brøndsted, Susana Casado, Pablo Cifuentes, John Deaton, Thomas G. Denes, Mohammad Aminul Islam, Rob Lavigne, Andrea I. Moreno-Switt, Natsuko Nakayama, Cristina Muñoz Madero, Alexander Sulakvelidze, Antonet M. Svircev, Jeroen Wagemans, Elena G. Biosca, Dácil Rivera

**Affiliations:** 1 Instituto de Productos Lácteos de Asturias—Consejo Superior de Investigaciones Científicas (IPLA-CSIC), 33300 Villaviciosa, Spain; pgarcia@ipla.csic.es; 2 Centro de Investigaciones Científicas y Tecnológicas de Extremadura (CICYTEX), 06071 Badajoz, Spain; rafael.tabla@juntaex.es; 3 Guelph Research and Development Centre, Agriculture and Agri-Food Canada, Guelph, ON N1G 5C9, Canada; hany.anany@agr.gc.ca; 4 Food Science Department, University of Guelph, Guelph, ON N1G 2W1, Canada; 5 Instituto de Biología, Pontificia Universidad Católica de Valparaíso, Valparaíso 2340000, Chile; roberto.bastias@pucv.cl; 6 Department of Veterinary and Animal Sciences, University of Copenhagen, 1870 Copenhagen, Denmark; lobr@sund.ku.dk; 7 Spanish Agency of Medicines and Health Products (AEMPS), 28022 Madrid, Spain; scasado@aemps.es; 8 PhageLab®, Santiago 7820244, Chile; pcifuentes@pht.cl; 9 ADM Science & Technology, Kennesaw, GA 30152, USA; john.deaton@adm.com; 10 Department of Food Science, University of Tennessee, Knoxville, TN 37996, USA; tdenes@utk.edu; 11 Paul G. Allen School for Global Health, Washington State University, Pullman, WA 99164, USA; amin.islam@wsu.edu; 12 Department of Biosystems, KU Leuven, B-3001 Leuven, Belgium; rob.lavigne@kuleuven.be (R.L.); jeroen.wagemans@kuleuven.be (J.W.); 13 Escuela de Medicina Veterinaria, Facultad de Agronomía y Sistemas Naturales, Facultad de Ciencias Biológicas, Facultad de Medicina, Pontificia Universidad Católica de Chile, Santiago 7820435, Chile; andrea.moreno@uc.cl; 14 Japan Fisheries Research and Education Agency (FRA), Hiroshima 739-0452, Japan; nakayama_natsuko37@fra.go.jp; 15 Department of Medicines for Veterinary Use, Coordinator of the National Antibiotics Plan, Spanish Agency of Medicines and Health Products (AEMPS), 28022 Madrid, Spain; cmunoz@aemps.es; 16 Intralytix, Inc., 8681 Robert Fulton Dr., Columbia, MD 21046, USA; asulakvelidze@intralytix.com; 17 Agriculture and Agri-Food Canada, Vineland Station, ON L0R 2E0, Canada; antonet.svircev@agr.gc.ca; 18 Departamento de Microbiología y Ecología, Universitat de València (UV), 46100 Valencia, Spain; 19 Escuela de Medicina Veterinaria, Facultad de Ciencias de la Vida, Universidad Andrés Bello, Santiago 8320000, Chile

**Keywords:** bacteriophages, antimicrobial resistance, sustainability, agrifood chain, One Health

## Abstract

The focus of this meeting was to discuss the suitability of using bacteriophages as alternative antimicrobials in the agrifood sector. Following a One Health approach, the workshop explored the possibilities of implementing phage application strategies in the agriculture, animal husbandry, aquaculture, and food production sectors. Therefore, the meeting had gathered phage researchers, representatives of the agrifood industry, and policymakers to debate the advantages and potential shortcomings of using bacteriophages as alternatives to traditional antimicrobials and chemical pesticides. Industry delegates showed the latest objectives and demands from consumers. Representatives of regulatory agencies (European Medicines Agency (EMA) and Spanish Agency of Medicines and Health Products (AEMPS)) presented an update of new regulatory aspects that will impact and support the approval and implementation of phage application strategies across the different sectors.

## 1. Introduction

Antimicrobial resistance presents an urgent challenge to global health, whose origin has been suggested to be in the misuse of these compounds for intensive livestock and agriculture practices. New strategies are crucial to combat undesirable bacteria in the agrifood sector while fulfilling the premise that they must be sustainable.

Bacteriophages are viruses of bacteria and are the natural enemies of bacteria by infecting and killing bacteria during phage propagation. Importantly, their ubiquity and diversity in all environments, including the human body, suggest that phages are harmless. Numerous studies have probed their potential to control pathogenic bacteria in several natural and human-made environments. The increase in bacteria resistant to multiple antibiotics has boosted the research on phages as antibacterials and the creation of companies intended to commercialize phage-based products. The aim of these activities is to provide novel solutions to stop the emerging growth and spread of antibiotic resistance worldwide. Moreover, restrictions in the use of antibiotics in raising livestock as well as in aquaculture have further pushed the need for novel antibacterial solutions. Strategies based on bacteriophages are promising and may indeed improve the resilience of food production systems by anticipating the risk of transmission of bacterial pathogens. In terms of environmental impact, the biocontrol of plant diseases using bacteriophages will reduce the contamination of soil and water caused by pesticides and other chemical biocides dangerous for natural ecosystems, as well as will provide new tools for maintaining agricultural productivity. Overall, reducing antibiotic use will help in safeguarding the environment and ensuring consumer safety.

The Organization for Economic Cooperation and Development (OECD) through their Cooperative Research Programme (CRP) is highly aware of the future challenges and focus on sustainability. In this context, we have organized the ECOPHAGE workshop, a Conference sponsored by the OECD’s Co-operative Research Program: Sustainable Agricultural and Food Systems, that addressed the CRP Theme II. Strengthening Resilience in the Face of Multiple Risks in a Connected World, and specifically the points “Antimicrobial resistance” and “Food Safety” [1,2]. The meeting discussed the use of bacteriophages as alternative antibacterials to achieve the sustainability of agrifood sector for future food demand and explored the possibilities and opportunities to implement this strategy in agriculture, animal husbandry, aquaculture, and food production sectors.

To the best of our knowledge, ECOPHAGE was the first workshop concerning the application of phages in the agrifood sector, gathering all involved stakeholders including phage researchers, agrifood industry representatives, and policymakers. By aiming for sustainable solutions, important benefits including a reduction in the release of antimicrobial residues into the environment and the subsequent decrease in antimicrobial resistance were achieved. This will further benefit the food production system in becoming safer and more efficient, reducing its environmental impact, and minimizing the transmission of multiresistant pathogens through the food chain and to the environment [1]; this will also contribute to the potential reduction in the risk of human antibiotic-resistant infections. This workshop offered the opportunity of discussion between the different stakeholders and presented to policymakers the complete state-of-the-art information to help them in their decision-making, seeking to contribute to the One Health approach and provide sustainable solutions for the future [1,2].

## 2. Scientific Program Conference, Speakers, and Roundtable Presentations

The ECOPHAGE workshop was held in Valencia, 12–13 September 2023 (Universitat de València), and the scientific program included 15 plenary presentations, divided in four sessions, and the corresponding round table for each session (Table 1). In addition, the CRP Theme Coordinator (Dr. Herman) provided a brief presentation about the OECD CRP. To close the event, a wrap-up session with a summary of the main conclusions was provided. The focus of this meeting assessed the suitability of the use of bacteriophages as alternative antimicrobials in the agrifood sector in the following sessions: Session I: Animal Production, Aquaculture and Veterinary; Session II: Agriculture and Environmental Issues; Session III: Improving Safety in Food Production; and Session IV: Citizen Science: Improving Consumer Perception of Phage-based Products. The invited speakers included four from the USA, three from Chile, two from Canada, two from Belgium, two from Spain, one from Japan, and one from Denmark.

## 3. Meeting Attendees

**Figure 1:**
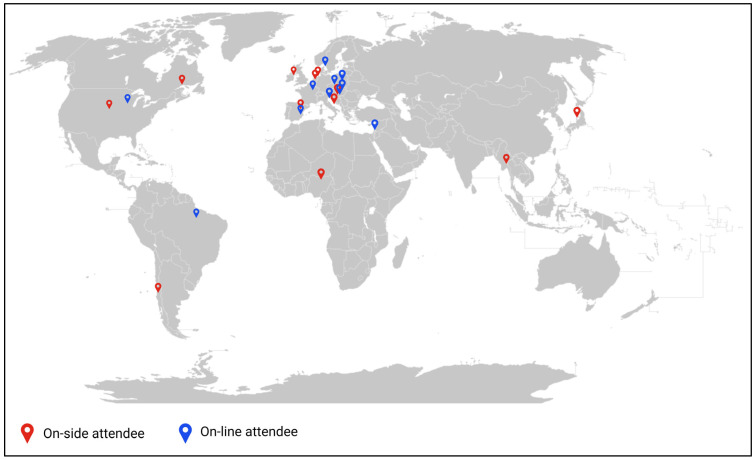
World map distribution of participants.

The ECOPHAGE meeting had a total of 105 participants, among them 74 were on-site and 31 were online and included researchers, predoctoral and postdoctoral students, and industry professionals from 19 countries in four continents (America, Europe, Africa, and Asia) (Figure 1). Furthermore, we wish to highlight the participation of representatives from 23 industries related to bacteriophages, biotechnology, food, agriculture, etc. Detailed information about the meeting can be found at https://www.ecophagevalencia2023.com/.

## 4. Scientific Sessions: Major Highlights from the Presentations

### 4.1. Session I: Animal Production, Aquaculture and Veterinary

The first session of the workshop, chaired by Dr. Rafael Tabla Sevillano (Dairy Department, Technological Institute of Food and Agriculture—Scientific and Technological Research Centre of Extremadura, INTAEX—CICYTEX, Badajoz, Spain) focused on phage applications to control animal pathogens. This topic was approached from the scientific, industrial, and regulatory point of view. The session started off with the keynote lecture by Dr. Andrea Moreno-Switt of the Pontifical Catholic University of Chile (Santiago, Chile), who is devoted to the mitigation of foodborne pathogens. *Salmonella* is a recognized foodborne and zoonotic pathogen that causes an estimated global burden of 93 million cases and 155,000 deaths [3]. While *Salmonella* is a highly diverse pathogen with more than 2600 serovars, only a few of these bacteria are commonly associated with human disease. Importantly, food animals and their products are among the most important sources attributed to foodborne outbreaks. Dr. Andrea Moreno-Switt’s work uses the distribution, diversity, and antimicrobial resistance of *Salmonella* serovars to identify the predominant strains of interest to target bacteriophage-based interventions. A collection of more than 2000 isolates from humans, animals, food, and water determined that multidrug-resistant *Salmonella* Infantis is the main strain to target for a phage intervention. Genomic analysis of 440 genomes of *S*. Infantis isolates found two clades of *S.* Infantis circulating in Chile since 2009 [4,5]. Overall, 92% of its strains clustered together and presented multiple antimicrobial resistance genes. To develop a precision phage cocktail for this strain of interest, a library of over 400 phages [4] was screened against a representative isolate of *S.* Infantis, identifying six specific phages. The selected phages were sequenced and characterized for their host range and lytic abilities. To design the phage cocktail, a panel of 10 *S.* Infantis isolates were used to identify the best phage combination [6]. Two models were successfully evaluated for challenge-phage assays: (i) an intervention model in chickens infected with *S.* Infantis that assessed a microencapsulated cocktail, which reduced 2 logs of *S.* Infantis; and (ii) an intervention model in chicken breast, which reduced *S.* Infantis below the detection limit of MPN/gr.

The next speaker was Dr. Roberto Bastías (Catholic Pontifical University of Valparaíso, Chile), who discussed their lab’s research pertaining to the use of phages as antimicrobials in aquaculture. The use of phages as antimicrobials has already been successfully evaluated with different species of cultivated fish and other marine animals, targeting pathogens such as *Flavobacterium columnare*, *F. psychrophilum*, *Aeromonas salmonicida*, *Vibrio parahaemolyticus*, *V. anguillarum*, among other bacterial species. Their research group has been involved in various trials assessing the use of phages as antimicrobials in aquaculture. In trials conducted on salmonids, they showed that phage CHOED was very effective in controlling *V. anguillarum* infections in fish, even with a multiplicity of infection of one [7]. Moreover, they demonstrated that the CHOED phage provided nearly 100% protection to salmon (*Salmo salar*) against *V. anguillarum* infection in aquaculture farm settings, showing a lower mortality in challenge aquarium tanks inoculated with the bacteria than in control aquarium tanks. Similarly, they participated in a study that showed that a phage mixture can reduce the *Vibrio* load in live feed used for gilthead seabream (*Sparus aurata*) cultivation by over 90% [8]. These trials revealed some of the challenges faced by this technology, such as managing phage-resistant bacteria. Results have shown that these resistant mutants can have alterations in their fitness or virulence, like a reduction in their growth rate, proteolytic activity, biofilm production, motility, or their general biochemical profile. However, these changes are not present in all cases [9]. Different strategies have been evaluated to address this issue, and the use of phage cocktails being the most common alternative. Another challenge associated with this technology is the selection of methods for phage administration, as this factor can affect the therapy’s effectiveness and must also adapt to various aquaculture production systems (tanks, open sea, or recirculation systems). In this regard, phages have been administrated orally with feed, with immersion, or even with injections [10]. Adding phages to the food allows a permanent presence of phages in the organs. As an alternative release system, the encapsulation of phages in alginate spheres was proposed. Regarding the regulatory framework for the use of phages in aquaculture, every country has different applicable regulations, so the phage-based products must be registered under a specific office. However, the lack of specific regulation for the use of phages as antimicrobials in most countries was highlighted, causing many companies to market these products as dietary supplements rather than antimicrobials.

An important topic for the phage therapy implementation was covered by Pablo Cifuentes MSc., co-founder and Chief Technology Officer of PhageLab^®^ (Chile), a company dedicated to the development of bacteriophage-based products for animal health. With the lecture “Phage application model in animal production systems”, they discussed the role of technology-based companies and the private sector in the maturation and readiness of the technology, where the demonstration in relevant operational environments is a key aspect of technology transference from the academia to the society. In that context, they exposed the development process of Milkeeper^®^, a feed additive that prevents infectious diarrhea in dairy calves caused by *Escherichia coli* and *Salmonella* spp. For this purpose, two field trials were executed at different scale levels in two different countries. In Chile, one farm of 20 calves was split into two groups, control and treatment. The product was applied every day during the first 60 days of life of the animals. At the end of the trial, the mortality rate decreased from 20% to 2%. Also, during the 60 days of trial, the morbidity rate was lower in the treatment group than the control group. The second trial was executed in Brazil with a total sample size of 200 calves. For this experiment, two farms were split into two sets, the control and treatment. The product was applied every day during the first 30 days of life of the animals. At the end of the trial, the Daily Weight Gain (DWG) was significantly improved from 0.26 to 0.35 Kg (*p* < 0.001). Since 2021, this powder formulation is distributed by MSD under the name of PhageIn in Latin-American countries, such as Chile, Argentina, and Brazil. This process allowed PhageLab to build a development platform for creating different products for different categories, like swine and poultry. For example, INSPEKTOR^®^ is a liquid feed additive that efficiently eliminates *Salmonella* spp. in broilers. The split farm challenge demonstrated that, at day 28, INSPEKTOR^®^ decreased the prevalence of *Salmonella* spp. from 80% to 20%, as well as the load of *Salmonella* spp. in swab samples. This product was also evaluated in a Clinical Research Organization to evaluate productive parameters and tolerance, concluding that the time to reach the slaughter weight was shorter in broilers and the weight gain was significantly higher in the INSPEKTOR^®^ group.

The session was concluded by Dr. Susana Casado (Head of Unit of Immuno-Bio Veterinary Medicines. AEMPS, Spain), who explained the regulatory framework of bacteriophage-based products for treating or preventing diseases in animals. Currently, bacteriophages are reappearing in the therapeutic arsenal as a potential alternative to antibiotic therapy (or to complement the latter), due to the increasing antibiotic resistance. These products are considered as Veterinary Medicinal Products (VMPs) and classified as medicines, so they require a marketing authorization before commercialization, as stated in Regulation (EU) 2019/6 of the European Parliament and of the Council of 11 December 2018 on veterinary medicinal products and repealing Directive 2001/82/EC [11]. Bacteriophages as therapies present certain peculiarities that pose scientific and regulatory challenges. Their action is linked to their lytic activity, generally restricted to specific bacterial strains. Additionally, the bacteriophage–host bacteria interaction is a dynamic process, and the host bacteria might develop resistance against bacteriophages to some degree. Consequently, VMP based on bacteriophages are expected to require frequent changes in their composition for the bacteriophage strain(s) in order to maintain efficacy/circumvent resistance development in relation to the intended indication. To avoid regulatory constrains due to the variable composition of bacteriophages as medicinal products, the European Medicines Agency-European Union (EMA) has published the Guideline for the quality, safety, and efficacy of veterinary medicinal products specifically designed for phage therapy (EMA/CVMP/NTWP/32862/2022) [12]. The guideline establishes the basis for the authorization of phage products with flexible qualitative and quantitative compositions as veterinary medicines. In general, the requirements stated in Annex II of Regulation (EU) 2019/6 [11] should be followed. Phage therapy products are classified as novel therapies (NT), and this allows the application of risk-based principles for determining the requirements for the marketing authorization. The allowed adaptation implies a deep scientific knowledge of the product, the application of risk-based approaches, and the appropriate design of quality risk management system and pharmaceutical quality systems. In the guideline, the concepts of parental bacteriophage products and representative preparations are explained. Both concepts allow the authorization of medicines with variable bacteriophage compositions. The guideline would benefit both the industry and regulators due to the provision of a relevant guidance on data requirements for bacteriophage-based medicinal products. It will facilitate and speed up the development and authorization of veterinary medicinal products specifically designed for phage therapy, hence, contributing to an increase in the availability of veterinary medicinal products tackling antibiotic resistance.

### 4.2. Session II: Agriculture and Environmental Issues

The second session of the Conference was chaired by Prof. Dr. Elena G. Biosca from the Microbiology and Ecology Department at the Universitat de València (Spain). It was focused on the use of bacteriophages as effective and safe plant control alternatives to protect economically important crops and to reduce the use of agrochemicals, including the use of antibiotics in non-European Union (EU) countries. Control of pathogenic bacteria through the use of free and immobilized lytic phages was also presented, as well as the algae-related virus application in environmental settings.

The first keynote speaker was Dr. Jeroen Wagemans (Laboratory of Gene Technology at KU Leuven, Belgium), an expert on bacteriophages and their applications, including the use of phages as biocontrol agents against several plant pathogenic bacterial species [13,14]. In his keynote lecture, he began the lecture by showing examples and advantages of phage-based biocontrol methods of globally important plant pathogenic bacteria such as *Agrobacterium*, *Erwinia amylovora*, *Ralstonia solanacearum*, and *Xylella fastidiosa*, and he compared the differences in the legislation and availability of commercial phage cocktails between the USA and the EU. Although, in the USA, there are several AgriPhage products registered for the control of different important phytopathogenic bacteria (Omnilytics) and, more recently a specific XylPhi-PD™ product for the control of *X. fastidiosa* in grapevine (A&P Inphatec), there are, to date, no registered phage cocktails available in EU countries. Next, he presented the results of bacteriophage biocontrol of the soilborne plant pathogen *Agrobacterium* responsible for hairy root disease in tomato production [15]. He explained the problem of hairy root disease caused by *Agrobacterium* biovar 1, an emerging disease in hydroponic greenhouses, and its current management method based on the use of hydrogen peroxide. Since the efficacy and sustainability of this agrochemical are in doubt because of the development of resistant strains, phages are successfully being explored as an alternative for the control of this plant disease. Thus, he presented the results of a recent study on the use of *Agrobacterium*-specific phages for the control of hairy root disease in hydroponic greenhouses [15]. Six *Agrobacterium*-specific bacteriophages were isolated from infected tomato greenhouse samples and characterized based on their structures and genomes. This allowed the identification of three different clades of bacteriophages, which were further evaluated for their ability to infect different *Agrobacterium* strains. Their efficacy to disinfect *Agrobacterium*-contaminated nutrient solutions was further evaluated, and the mutations responsible for phage resistance were identified. The stability of phages in greenhouse-relevant conditions was also evaluated. Finally, three phages were selected for further bioassays. Ongoing greenhouse biocontrol trials in potted tomato plants inoculated with the pathogen and treated with the selected phage cocktail revealed significant reductions in symptom development. Another semi-commercial greenhouse trial with plants on rockwool mats also showed the presence of significantly less symptomatic plants for the phage-treated object. Thus, Dr. Wagemans concluded that the selected phage cocktail could be a valuable tool for both nutrient solution decontamination and biological control of hairy root disease in a greenhouse setting.

The second keynote lecture was given by Dr. Antonet Svircev, a research scientist (Agriculture and Agri-Food Canada in Vineland Research Station in Ontario, Canada) and renowned expert in microbiology, plant pathology, and phage-based control methods of fruit tree pathogens. She presented on the applications of phages for agriculture with a focus on the control of fire blight, a globally devastating disease of apple and pear trees caused by the bacterial pathogen *Erwinia amylovora*. She began her lecture by providing an overview of the problem of the emergence of antibiotic resistance in apple trees, as well as the society’s demand for safe fruits, which has prompted the development of phage-based methods for the biocontrol of *E. amylovora* [16,17]. Next, she presented bioprospecting and selecting lytic phages against *E. amylovora* with a wide range of hosts including their characterization, followed by the development of a biocontrol method by exploiting the synergy of using an epiphytic bacterium of apple trees with antagonistic activity against this pathogen, *Pantoea agglomerans*, as a carrier of the *E. amylovora* phages to the flowers [18]. This bacterial carrier allows the propagation of *E. amylovora* phage populations on the stigma, improving the efficacy of a phage cocktail that infects and kills both bacterial species. Further, she explained how to study phage–host interactions to choose the right phages by the development of quantitative PCR (qPCR) with a standard plasmid for the simultaneous detection and quantification of *E. amylovora*, the bacterial carrier and phages from four different genera, as well as to determine the host range of phages on a collection of *E. amylovora* strains from all over the world [19]. The genomic study of this global collection allowed to determine most of the strains belonging to the North American widely prevalent (WP) clade [17]. Combining these data with those of host range, together with the knowledge of capsular exopolysaccharides, allowed the best phages to be selected [19]. In addition, since for the development of phages as biological products, it is necessary to understand the mechanisms of bacterial resistance to phages; thus, global *E. amylovora* and *P. agglomerans* populations were screened for lysogens. Parcey et al. [20] designed and tested an artificial CRISPR-Cas system and demonstrated that it is not an efficient phage resistance system in preventing myovirus infections. Finally, results from field trials using a formulated and large-scale-produced phage carrier demonstrated that an effective statistically significant biocontrol can be achieved with phages, but this efficacy is highly dependent on seasonal weather conditions [21]. Overall, Dr. Svircev’s lecture helped in understanding the landscape of *E. amylovora* phages, from their discovery and characterization to their journey to become a biological tool for eco-sustainable agriculture and safe food.

The following keynote lecture was presented by Dr. Hany Anany (Agriculture and Agri-Food Canada and University of Guelph, Canada), a senior scientist involved in several research projects related to the use of bacteriophages to improve food safety with a focus on novel ecofriendly control and detection methods that can be applied to foodborne and plant pathogens. His presentation was on the biosanitation and biocontrol potential of free and immobilized bacteriophages to enhance the safety of food products by reducing bacterial contamination in food processing settings, as well as to enhance the phage storage stability and shelf life [22,23]. He introduced how outbreaks of foodborne diseases and the growing demand for safer foods have prompted the search for natural alternatives to control pathogenic bacteria. Phages have proven to be an alternative with different applications in the treatment of animal and plant bacterial diseases, as well as in the reduction of bacterial biofilms and contamination in food processing settings [23,24]. In his lecture, Dr. Anany presented examples of using phages to improve safe food production, including phage encapsulation and immobilization methods to improve phage delivery and storage stability [25]. In the first case, he showed the results of biosanitation assays using free lytic phages that were able to disperse *Listeria monocytogenes* biofilms by mimicking dairy processing conditions. A 2-log reduction in bacterial counts one week after the addition of the selected phages was achieved. The second example was the encapsulation of the bacterial carrier of *E. amylovora* phage cocktail developed by Dr. Svircev’s team, *P. agglomerans* infected with these phages, to improve the phage storage stability as well as the phage shelf life under field conditions [26]. The third one was focused on the formulation of a cocktail of five *Salmonella* phages, which recognized different host receptors. These phages were immobilized on a new material membrane and their release was monitored in food processing settings to reduce the risk of this pathogen. The immobilized phages maintained their activity at different temperatures, and their application to artificially contaminated raw chicken reduced *Salmonella* counts by 2 log after one day. Results from genomic and phenotypic characterizations of *Salmonella* and *L. monocytogenes* bacteriophage-insensitive mutants (BIMs) were also depicted, as well as the phage-based biosensors for the rapid detection of foodborne pathogens in different food matrices [27]. Thus, the results presented by Dr. Anany demonstrated that phages can be used as biosanitizing agents in food processing facilities. They can also be encapsulated to improve the phage storage stability and delivery in fruit orchards. Further, phages can be immobilized to extend their applications, such as reducing the risk of foodborne pathogens in poultry packaging materials. All cases contribute to improving food safety in an ecofriendly manner.

The last keynote speaker of the second session was Dr. Natsuko Nakayama (Japan Fisheries Research and Education Agency), Head of the Harmful Algal Blooms Group at the National Research and Development Agency of Japan. She is an expert in the ecology of marine viruses, phages in rice paddies, and biocontrol against harmful algal blooms in marine settings using viruses, as well as in host–virus interactions and viral diversity. In her lecture, she provided information on the current environmental status of oyster farms in Lake Kamo in Japan and on the viral control of the harmful proliferation of marine microalgae *Heterocapsa circularisquama*, which is related to light, slow-moving water and water nutrients. *H. circularisquama* is harmful to shellfish such as oysters, pearl oysters, and clams, and, in recent years, its blooms have been gradually expanding, thereby causing serious damage in coastal waters and oyster farms in Japan [28]. Thereafter, she talked about *H. circularisquama* RNA virus (HcRNAV), a single-stranded RNA virus that infects *H. circularisquama* and can be genetically divided into at least three groups [29,30]. She showed that the abundances of these toxic microalgae and their infecting HcRNAV are closely correlated each year, and that these HcRNAVs accumulate in the sediment at the end of the blooms. She then explained the advantages of using HcRNAV as a natural agent to control *H. circularisquama* populations in the environment, with a high host specificity and significant increases in its populations as it destroys the host cell. Thus, she discussed a viral control method using sediments containing HcRNAV, as well as the difficulties in convincing oyster farmers to introduce viruses directly into the environment, as viruses make a negative impression on the local society. However, once she obtained the required government authorizations, a sediment dispersal trial of HcRNAV in Lake Kamo was carried out using indigenous sediments with HcRNAV from multiple locations and local periods for a more effective application. This method was shown to be successful in reducing *H. circularisquama* proliferation in environmental settings [28]. In summary, Dr. Nakayama showed that the virus-based control of *H. circularisquama* offers an effective, specific, and safe solution to control Heterocapsa algae blooms, with minimal environmental impact and increased efficiency compared to traditional methods. However, for an efficient implementation of this bloom control strategy, it is necessary to pool autochthonous sediments containing HcRNAV from multiple local sites and periods.

### 4.3. Session III: Improving Safety in Food Production

The third session on application of phages in food, chaired by Dr. Pilar García (IPLA-CSIC, Spain), began with a keynote lecture by Dr. Mohammad Aminul Islam (Paul G. Allen School for Global Health, Washington State University, Pullman, WA, USA) who discussed the findings of a molecular epidemiological study that was designed to identify the sources and transmission routes of multidrug-resistant *E. coli*, especially the extended spectrum beta lactamase-producing *E. coli* (ESBL-Ec) that causes community-acquired urinary tract infections (CA-UTI). Indeed, multidrug-resistant (MDR) uropathogenic *E. coli* (MDR UPEC), which causes urinary tract infections (UTI), is presumed to have originated in poultry and transmitted to humans via the food chain. By sampling both community-acquired UTI patients and high-risk food sources contemporaneously in Dhaka, Bangladesh, the phylogenetic relatedness among the isolates were identified. During 2017–2018, a total of 2297 food samples including raw poultry meat, fresh-produce, raw fish, raw eggs, and raw beef samples collected from 23 wet-markets in Dhaka were tested for ESBL-Ec. During the same time-period, 112 ESBL-Ec strains isolated from 394 CA-UTI patients, living in the same areas from where food samples were collected, were included for comparative analysis. Phylogroups of food and clinical ESBL-Ec were identified, and the isolates belonging to the same phylogroups were analyzed using whole genome sequencing (WGS). About 26% (n = 600) food samples were positive for ESBL-Ec. Poultry meat was predominantly positive for ESBL-Ec followed by raw beef, raw fish, eggs, and fresh-produce. The majority of clinical ESBL-Ec (67%) belonged to phylogroup B2, but none of the food isolates belonged to this phylogroup. Phylogroups A, C, D, E, and F were commonly found in both sources. WGS analysis revealed that except for three isolates, all ESBL-Ec from food sources were phylogenetically unrelated to ESBL-Ec from CA-UTI patients. Furthermore, MDR UPEC isolates in humans are highly clustered in a few genetic lineages that are uncommon in food isolates, indicating that MDR UPEC are less likely to have originated from food sources [31]. Overall, Dr. Islam’s research focuses on understanding the contribution of food sources in the emergence and transmission of MDR *E. coli* that causes extra-intestinal infections, with a long-term goal of identifying effective and scalable interventions to reduce the burden of AMR infections in resource-poor settings.

The next keynote speaker was Dr. Thomas Denes (Department of Food Science, University of Tennessee, USA), who presented his research addressing challenges that can impact the application of phages to remove *Listeria monocytogenes* from food. This bacterium is an opportunistic foodborne pathogen and causative agent of listeriosis (mortality rate between 15–30%). Approximately, 1600 illnesses and 260 deaths related to *Listeria monocytogenes* are recorded every year in the US. Phage-based products are currently in use in the United States and globally to combat the foodborne pathogen *L. monocytogenes*. However, their efficacy might be constrained by two critical challenges. Firstly, the development of phage resistance is predictably selected under the pressure of lytic phages. Secondly, the complexity of various food matrices may restrict the lytic capabilities of phages. Dr. Denes explores the potential of directed evolution as a strategy to mitigate these hurdles, particularly in relation to phages infecting *L. monocytogenes*, by selecting *Listeria* phages capable of countering phage resistance. Previous work showed that phages LP-125 and LP-048 consistently select phage-resistant *L. monocytogenes* with mutations in genes affecting wall teichoic acids and that these mutations were confirmed to affect phage adsorption. Dr. Denes’ team identified a wild-type *Listeria* phage, LP-018, that is able to form plaques on a phage-resistant *L. monocytogenes* strain lacking rhamnose in its cell wall’s teichoic acids [32,33]. This phage may be useful in biocontrol applications that aim to reduce the emergence of phage resistance. Short-term coevolution experiments allow to investigate the impact of single phages and a two-phage cocktail on the regrowth of phage-resistant *L. monocytogenes* and the adaptation of the phages to overcome this resistance. Infections with *Listeria* phages LP-048, LP-125, or a combination of both select different populations of phage-resistant *L. monocytogenes* bacteria with different regrowth times. Phages isolated from the end of the coevolution experiments were found to have gained the ability to infect phage-resistant mutants of *L. monocytogenes*. Interestingly, phages isolated from coinfected cultures were identified as recombinants of LP-048 and LP-125 [34]. This strategy can be utilized to obtain mutant and recombinant phages with adapted host ranges, and then, these phages may be useful for limiting the emergence of phage resistance and for targeting strains that show general resistance to wild-type phages. Additionally, he showed that a similar directed evolution approach can be used to select mutant *Listeria* phages with enhanced binding efficiency in milk conditions [35].

The third presentation was given by Dr. Alexander Sulakvelidze (Intralytix, Inc., Columbia, SC, USA) on the phage-based control of bacterial pathogens in environmental and food processing settings. Foodborne illnesses remain a major cause of hospitalization and death worldwide despite many advances in food sanitation techniques. Traditional antimicrobial methods, such as pasteurization, high pressure processing, irradiation, and chemical disinfectants, have considerable drawbacks, such as a large initial investment, potential damage to processing equipment due to their corrosive nature, and a deleterious impact on the organoleptic qualities and nutritional value of foods. Moreover, these strategies kill indiscriminately, including many beneficial bacteria that are naturally present in foods. One promising technique that addresses several of these shortcomings is bacteriophage biocontrol, a green and natural method that uses lytic bacteriophages to specifically target pathogenic bacteria in foods [36]. Phages are naturally present in all fresh unprocessed foods, and they have been isolated in prodigious numbers from a wide range of various food products, including ground beef, pork sausage, chicken, farmed freshwater fish, common carp and marine fish, oil sardines, raw skim milk, and cheese. Lytic phages exhibit a remarkable bactericidal potency against their specific bacterial host strains. Because of their potent, highly specific antibacterial activity, phages provide an all-natural, nontoxic, and effective means for targeting bacterial pathogens present in various foods as well as on surfaces of food processing facilities and other food handling establishments. Several phage-based products have been recently introduced, including ListShield™—the first ever phage-based product (developed by Intralytix, Inc.) to have received the Food and Drug Administration (FDA) approval for direct food applications. Most commercial phage-based food safety products are GRAS-affirmed by the FDA and listed as processing aids (no labeling required) by the USDA. Numerous recent scientific publications, as well as unpublished data from various commercial food processing facilities utilizing phage biocontrol as part of their Hazard Analysis Critical Control Point (HACCP) protocols, strongly suggest that phages, when properly applied, can significantly (typically by 1–3 logs) reduce the levels of their targeted bacterial pathogens in foods, thus making those foods safer for human consumption [37,38]. These phage preparations are typically simply sprayed on various foods or food contact surfaces in food processing facilities, although some other application methods (e.g., dip) have also been employed. The data also suggest that the efficacy of phage applications can vary considerably from one food processing facility to another. Two factors play a critical role: (1) the formulation of phage preparation (phage cocktails almost inevitably show a better efficacy compared to single phage preparations, and some commercial phage products are more effective than others), and (2) application methods (optimizing phage delivery physio-kinetics and developing a custom application system to ensure an optimal phage application to various types of foods is critical for achieving better efficacy vs. reduced efficacy/increased cost).

The third session was closed by Dr. John Deaton (ADM Science & Technology, Georgia, USA), who discussed about the microbiome and the role of bacteriophages in this environment. Recent studies have identified 142,000 viral species living in the human gut, after analyzing 28,060 human gut metagenomes from across the world. Also, the microbiome plays an important role in human health and whose balance is essential in a multitude of biological processes including inflammation and aging. Bacteriophages are ten-fold more abundant than bacteria in the gut; therefore, they are key modulators of bacterial populations. An important role related to non-host-derived immunity was also attributed to phages. Due to their harmlessness, phages have been proposed as probiotics for manipulating oral or gut microbiota. In a clinical study, 32 participants ingested a capsule for 28 days, which contained PreforPro^®^ (15 mg), a mixture of four bacteriophages. Results showed that the product was both safe and tolerable in a target human population of healthy individuals reporting moderate GI distress. In the PHAGE study, the effects of supplemental bacteriophage intake on inflammation and gut microbiota were analyzed in healthy adults. A significant change in microbiome with PreforPro was observed with a decrease in harmful bacteria and an increase in beneficial bacteria such as *Eubacterium dolichum*, *Eubacterium biforme*, and *Bifidobacterium bifidum*. Additionally, the study revealed that no changes in cytokine levels were observed pre- and post-placebo administration. There was a significant reduction in circulating IL-4 in the population taking PreforPro^®^ relative to the placebo [39]. Furthermore, the PHAGE-2 study demonstrated that supplemental bacteriophages extend *Bifidobacterium animalis* subsp. *lactis* BL04’s benefits on gut health and microbiota in healthy adults. 66 participants were recruited to consume placebo, *B. animalis lactis* BL04 (1 Billion CFU), and *B. animalis* lactis BL04 (1 Billion CFU) plus PreforPro^®^ (15 mg) for four weeks. Results showed a larger increase in *Lactobacillus* and *Bifidobacterium* and short chain fatty acid-producing microbial taxa. Also, a decrease in bacteria often associated with increased gut discomfort and imbalances, such as *Citrobacter*, *Desulfovibrio*, and *E. coli.*

### 4.4. Session IV: Citizen Science: Improving Consumer Perception of Phage-Based Products

The World Economic Forum listed the use of bacteriophages as one of the top 10 Emerging Technologies of 2023 [40]. The report highlights technologies that will have a positive impact on society in the next 3 to 5 years. Phages show a potential to treat diseases that affect human, animal, and plant health. They can also be used as feed supplements that improve livestock growth or eliminate pathogenic and zoonotic bacteria in the food supply chain, in line with the One Health approach. This demonstrates the high expectations for the development of phages as biocontrols of pathogenic bacteria, and, additionally, the commercialization and market utilization of phage formulations and therapeutics are expected to increase in the coming years, with an estimated annual growth rate of 8.1% from 2021 to 2028 [41].

The challenges that need to be addressed to improve the understanding of phage use are as follows: (a) The population’s perception of risk regarding phage use; for example, the study published by McCammon et al. [42] showed that there is a greater acceptance of phage therapy when it is described without using the word “virus”, using instead the phrase “natural bacterial predator”. This information may be especially relevant in the wake of the COVID-19 viral pandemic. Similarly, there is a positive preference for therapies approved for use in a Western European country, compared to an Eastern European country. There is a Western-European bias present in medical treatments. Therefore, this information can be used to influence the marketing and advertising of phages to maximize their uptake [42]. It was also concluded by this study that shorter treatment durations are preferred and always compared to antibiotic treatment times, which are often shorter. But the low side effects together with the high specificity to eliminate pathogenic bacteria without eliminating beneficial bacteria from the human body are among the positive characteristics of phase use [42]; (b) In terms of regulatory obstacles, a regulatory pathway must be sought that can incorporate the application of phages considering that it is not a drug; therefore, independent international organizations, such as Phages for Human Applications Group Europe (P.H.A.G.E), have been able to gain the support of phage biologists, physicians, and policymakers to recognize that phage therapy is a valid and promising option to treat bacterial infections in humans, but it is certainly only the beginning of a long and challenging road [43]; (c) Need for replacement of antibiotic and chemical treatment must be considered as an increasingly real possibility, due to the large silent epidemic of bacterial resistance, requiring action for the controlled use of antibiotics, together with the challenge of replacing pesticides by new technologies (EU (91/414/EEC and (EC) 889/2008) [44]. Pirnay et al. [45] described a magistral approach as a model for regulating phage therapy, in which phages are considered as active ingredients instead of industrially manufactured drugs. This approach, which is currently implemented in Belgium, involves a collaboration between phage manufacturers (these can be individual hospital pharmacies) and authorized Belgian laboratories to ensure the production of high quality, customized phages with targeted application for each patient, using biobanks following the current specified standards; and (d) Need to replace or complement the current forms of phage-based dissemination. On the other hand, because the awareness of phage therapy is low, it is necessary to consider how phages can be presented in a way understandable to the society at large. In this regard, it should be considered that, when awareness of a topic is low, errors in understanding and response are introduced. In these situations, the way the treatment is communicated to the public can have a profound impact on acceptance. For example, studies investigating vaccine-related dissemination show that media reporting on vaccine safety can influence public perceptions and, ultimately, acceptance [42].

Recently at ECOPHAGE, the session “Citizen Science: Improving Consumer Perception of Phage-based Products” was moderated by Dr. Dácil Rivera, PhD in Food Nutrition, and assistant professor at Andres Bello University of Chile, with the participation of the following experts: Dr. Lone Brøndsted, Professor in Phage biology and Biocontrol for food safety at the University of Copenhagen, Denmark; Dr. Rob Lavigne, scientific disseminator and chair member of the International Committee for Taxonomy of Viruses (ICTV) on the taxonomy of bacterial viruses and currently at Laboratory of Gene Technology/Department of Biosystems/KU Leuven, Belgium; and Dr. Cristina Muñoz, Head of the Preclinical and Clinical Area and Coordinator of the National Antimicrobial Plan (PRAN) of the Spanish Agency of Medicines and Health Products.

Dr. Lone Brøndsted presented novel alternatives for controlling zoonotic pathogens using phages and phage-derived technologies, both for tackling unwanted bacteria in animals and food. Lone presented three novel alternatives based on phage technology. The first is a collection of *Escherichia coli* phages capable of infecting extended-spectrum β-lactamase (ESBL) *E. coli* that is commensal in livestock [46] but poses a risk to humans by transferring antibiotic resistance to pathogenic strains. Thus, a One Health approach targeting ESBL has been already proposed in farm animals for the preventive use of phages. In the case of other foodborne pathogens like *Salmonella*, a large-scale industrial trial using phages targeting this pathogen in a slaughterhouse was presented [47]. The study showed that phage applications reduced *Salmonella*-positive carcasses by approximatively 79%. In addition, it demonstrated the feasibility and considerations for phage applications in industrial settings as an additional strategy to control foodborne pathogens. Finally, she presented a novel biotechnological solution to control *Campylobacter* in chicken meat products [48]. The novel antibacterial was designed by fusing phage receptor-binding protein of a prophage to endolysin of phage T5. This novel enzyme, called Innolysin, kills *Campylobacter* by degrading its peptidoglycan, resulting in a 1.6 log reduction of this foodborne pathogen on chicken skin during storage in conditions relevant for meat products. This presentation demonstrated the successful application of phages for controlling zoonotic pathogens involving specific solutions for each bacterium and developed according to the food matrix as well as the industrial setting.

Dr. Rob Lavigne gave an interesting presentation on the importance of phage applications and the impact of communication towards different stakeholders. These stakeholders include fellow scientists, people in industry and legislature, and the public. Generally, their interests are aligned as they all require information on the advantages of phage use and safety, considering the fact that several traditional chemical pesticides are being withdrawn from the European market in the context of achieving a more sustainable agriculture under EU regulatory requirements (91/414/EEC and (EC) 889/2008) [44]. In this regard, phage applications still face key hurdles that require critical input from the scientific community through the dissemination of high-quality research to guide legislative, economical, and practical implementation aspects of the phage biocontrol approval process. This communication provides an overview ranging from the efficacy and environmental safety of phage biocontrol to their economic and social issues, as societal communication requires education and proactive outreach [42]. Finally, he makes it clear that, as scientists, we need to go beyond traditional publication and patenting strategies for phage-based biocontrol to become a reality. This implies a stratified and sustained communication effort towards the different stakeholders that require the development of a different language than the one we usually use for the transmission of scientific knowledge. However, this societal communication requires education and proactive outreach. As current examples of outreach projects, the ‘Innovirology’ initiative led by Dr. Esperanza Gómez-Lucía [49], was presented. This consortium has published handbooks, games, and lectures, including ‘Virology: an interactive guide’ [50], and conducts tailored, local outreach. For example, the initiative called “Innovirology, a meeting place with drawing and science” held at the Complutense University of Madrid allowed the active integration of critical scientific literacy along with innovations in the teaching of STEM subjects. It helps to train teachers and virology researchers so that they gain new capabilities to transmit innovative educational content to students through attractive teaching programs and improved information platforms for the public. Innovirology made it possible to combine interactive podcasts, such as TWiV, for the transmission of evidence-based scientific knowledge in today’s digital and virtual era, in a language accessible to general audiences of all ages [49].

The last presentation was given by Dr. Cristina Muñoz, who presented a gap analysis by reviewing the regulatory tools and measures currently in place to support the development and regulation of alternatives to antimicrobials (ATAm) in veterinary medicine, with special emphasis on alternatives to antibiotics, and identifying where and how they could be improved. Some products containing the same active substance could be classified as veterinary medicinal products (VMPs), feed additives, or biocides, depending on the intended use and claims made. These products are often referred to as “borderline products”. The classification of the product will determine the appropriate regulatory framework and the standards against which it will be evaluated by the EMA. The use of alternatives to antimicrobials (ATAm) represents an avenue to reduce the use of antimicrobials, particularly antibiotics, in veterinary medicine. Therefore, there is a need to explore ways to recognize the importance of antimicrobial alternatives to reduce the need for antimicrobial use, particularly conventional antibiotics, in veterinary medicine and to take a proactive approach to promote their authorization.

Difficulties in the classification of certain products and the lack of clear guidance on general and specific technical requirements are perceived as the main challenges for the authorization of ATAm as veterinary medicinal products. To support developers, it would be useful to identify the provisions of the new Regulation (EU) 2019/6 and its Annex II that are applicable to ATAm (Regulation (EU) 2019/6, Annex II) [11]. It should be noted that, for some ATAm (e.g., vaccines and phages), the legal framework is well established, and appropriate guidance is available (www.ema.europa.eu, accessed on 1 October 2023). The European Regulatory Network has given high priority to activities that promote the availability of VMPs that can be used as alternatives to antimicrobial products, so depending on the active substance and mode of action, a “novel therapy” veterinary medicinal product can fall into any of three product categories: (a) veterinary medicinal products other than biologicals; (b) biological veterinary medicinal products other than immunologicals (e.g., bacteriophages V.1.5.4. in Annex 2); and (c) immunological VMPs. Therefore, the perceived challenges are as follows: (1) to include new criteria for assessing the level of efficacy that is different from that used for conventional antibiotics; (2) to evaluate target species for tolerance; (3) to explore designs for preclinical and clinical efficacy studies that allow the search for other parameters for comparison than antimicrobials; (4) studies of potential reduction of antimicrobial use in the benefit/risk assessment of ATAm; and (5) the need to develop case-by-case studies.

The ECOPHAGE workshop concludes that phage therapy is a technology that will continue to develop; however, for this development to be sustainable over time, efforts must be concentrated by scientists to consider the application not only as a specific treatment, but also as a biological intervention in biological systems (animals, environments, plants, or humans), which will require the study of multiple variables that may influence the success of the use of phages. In terms of scientific dissemination, the access to information for all types of people should be prioritized, through the use of new technological resources that allow generating active platforms of knowledge to limit negative speculations and, on the other hand, to promote scientific meetings that consider the experience of countries that have been successfully using phage therapy for decades such as Georgia, Poland, Russia, Belgium, the United Kingdom, among others, which have developed good initiatives such as Citizen Science Phage [51] that characterize the phages collected by the public from the environment. Targeting schools and science fairs to recruit “phage hunters” brings phage therapy closer to common knowledge. Not only does this expose the public to phages in a positive and engaging way, but also because of the abundance of phages in the natural environment, this approach provides a low-cost and replicable template to accelerate the development of phage libraries globally [42]. And in terms of regulations, current and flexible regulations should be developed that consider the nature of phages for proper application and monitoring of their effect.

## 5. Major Outcomes and Conclusions in Terms of Policy Relevance

This meeting highlighted several ideas about the future of bacteriophages as antimicrobials in the agrifood sector and the challenges that need to be resolved. The main conclusions of this review are as follows.

The efficacy of phages to remove undesirable bacteria from the different sectors has been widely demonstrated. However, the application for each individual case would require more research to adapt specific phage preparations. For instance, proper formulations and delivery strategies, the design of cocktails using Artificial Intelligence (AI), and the control of phage-resistant target bacteria are some issues to consider.

We conclude that phages may be used to reduce the need for the use of antibiotics and chemical pesticides, both as prophylactic agents and treatments of animal and plant infectious diseases and zoonosis. Additionally, phages have been recently proposed as probiotics to modify the intestinal microbiota by decreasing harmful bacteria and increasing the beneficial ones. Also, endolysins (phage lytic enzymes) remain a promising field with applications in both human and animal health.

An important point is regarding phage safety assessment. No major environmental risks or toxicities are expected due to the wide distribution of phages in all environments, but this needs to be validated to meet regulatory requirements, as a consequence of the wide diversity of applications and the different regulations adapted to each specific setting (animal, environmental, and plant contexts). Fortunately, regulatory agencies such as EMA and FDA are working on this subject.

To achieve these objectives, proper communication between scientists, industry, society, policymakers, and risk assessors is essential. The implementation of suitable solutions against antibiotic resistance is a very complex problem that requires further research and a transfer to industry, but also an adoption by consumers and farmers, driven by tailored communication and supported by customized regulations.

## Figures and Tables

**Table 1:** Scientific program schedule.

Session Name	Participation Role: Moderator (M) or Speaker (S)	Workplace and Participant’s Category, Country	Activities and Topic Number (Tx)
Welcome, Opening Session	Pilar Garcia (M)	Director of Ecophage. Instituto de Productos Lácteos de Asturias. Consejo Superior de Investigaciones Científicas (IPLA-CSIC), Spain	Brief presentation about the OECD Cooperative Research Programme (CRP) Coordinator
Opening Remarks	Authorities of Universitat de València, Spain	Institutional representatives from the Universitat de València, Spain	Coordinator and organizer’s presentation
**Session I: Animal Production, Aquaculture and Veterinary.**	**Rafael Tabla (M)**	**Organizer of ECOPHAGE, Centro de Investigaciones Científicas y Tecnológicas de Extremadura (CICYTEX), Spain**	**Presentation of Session I**
	Andrea Moreno-Switt (S)	Research Center/University. Catholic Pontifical University of Chile.	T1: One Health approach to study *Salmonella* to develop a precision bacteriophage-based intervention for food safety.
	Roberto Bastias (S)	Research Center/University. Catholic Pontifical University of Valparaíso, Chile.	T2: Use of bacteriophages for the control of diseases affecting farmed fish.
	Pablo Cifuentes (S)	Companies. PhageLab, Chile.	T3: Phage application model in animal production systems.
	Susana Casado (S)	Policy regulators. Spanish Agency of Medicines and Health Products (AEMPS), Spain.	T4: Guideline on quality, safety, and efficacy of bacteriophages as veterinary medicine.
	Rafael Tabla (M)	Organizer of Ecophage, CICYTEX, Spain.	Roundtable N°1
**Session II: Agriculture and Environmental Issues.**	**Elena G. Biosca (M)**	**Organizer of Ecophage.** **Universitat de València, Spain**	**Presentation of Session II**
	Jeroen Wagemans (S)	Research Center/University. University of Leuven, Belgium.	T5: Back to the roots: phage biocontrol of *Agrobacterium*, causing hairy root disease in tomato production.
	Antonet M. Svircev (S)	Research Center/University. Agriculture and Agri-Food, Canada.	T6: Bacteriophages applications for eco-sustainable agriculture with focus on the control of fire blight.
	Hany Anany (S)	Research Center/University. Agriculture and Agri-food, University of Guelph, Canada.	T7: Bio-sanitation and Bio-control potential of free and immobilized bacteriophages to enhance food safety.
	Natsuko Nakayama (S)	Research Center/University. Japan Fisheries Research and Education Agency (FRA), Japan.	T8: Application of a virus-based control method reducing the damages by the marine harmful dinoflagellate *Heterocapsa circularisquama.*
	Elena G. Biosca (M)	Organizer of Ecophage. Universitat de València, Spain.	Roundtable N°2
**Session III: Improving Safety in Food Production.**	**Pilar García (M)**	**Director of Ecophage.** **IPLA-CSIC, Spain**	**Presentation of Session III**
	Mohammad Aminul Islam ^1^ (S)	Research Center/University. Paul G. Allen School for Global Health, Washington State University, USA.	T9: Antimicrobial resistance among human, plant and animal sources: understanding the transmission risks in hot spots.
	Thomas Denes (S)	Research Center/University. University of Tennessee, Knoxville, USA.	T10: Overcoming challenges for phage applications in food safety with directed evolution.
	Alexander Sulakvelidze (S)	Companies. President and CEO Intralytix, Inc., USA.	T11: Phage-based control of bacterial pathogens in environmental and food processing settings.
	John Deaton (S)	Companies. ADM Science & Technology, Georgia, USA.	T12: The future of bacteriophages in industry.
	Pilar García (M)	Director of Ecophage. IPLA-CSIC, Spain.	Roundtable N°3
**Session IV: Citizen Science: Improving Consumer Perception of Phage-based Products.**	**Dácil Rivera (M)**	**Organizer of Ecophage.** **University Andres Bello, Chile**	**Presentation of Session IV**
	Lone Brøndsted ^1^ (S)	Research Center/University. University of Copenhagen, Denmark.	T13: Advancing phage applications in food production
	Rob Lavigne (S)	Research Center/University. University of Leuven, Belgium.	T14: Importance of scientific dissemination in the risk perception of bacteriophage use.
	Cristina Muñoz Madero (S)	Policy regulators. Department of Medicines for Veterinary Use. Coordinator of the National Antibiotics Plan. AEMPS, Spain.	T15: Solving doubts about the legislative framework for new antimicrobials.
	Dácil Rivera (M)	Organizer Ecophage. Universidad Andrés Bello, Chile.	Roundtable N°4
Wrap-up session	Dácil Rivera (M)	Organizer Ecophage. Universidad Andrés Bello, Chile.	Summary and closing session of the workshop through a dynamic activity with the audience.

^1^ These researchers presented their topic online.
